# Profile of usage of a reference diagnostic service on oral pathology: a 10-year evaluation

**DOI:** 10.1186/s12913-014-0653-7

**Published:** 2014-12-20

**Authors:** Karla Rachel Oliveira e Silva, Ana Luísa Lara Siqueira, Patrícia Carlos Caldeira, Mauro Henrique Nogueira Guimarães de Abreu, Maria Cássia Ferreira de Aguiar

**Affiliations:** Department of Oral Pathology and Oral Surgery, School of Dentistry, Universidade Federal de Minas, Gerais, Brazil; Department of Community and Preventive Dentistry, School of Dentistry, Universidade Federal de Minas, Gerais, Brazil

**Keywords:** Oral pathology, Oral diagnosis, Public Health, Epidemiology

## Abstract

**Background:**

Despite the professional and academic relevance of the Brazilian oral pathology diagnostic laboratories, no information about their usage profile is available in the English literature. The objective of the present study is to report data about the histopathological and immunohistochemical exams performed in a Brazilian regional reference laboratory of oral pathology, as well as its main users.

**Methods:**

Information about all histopathological exams performed between 2002 and 2012 was retrieved from the files of the Oral and Maxillofacial Pathology Service of the School of Dentistry of Universidade Federal de Minas Gerais. Data collected included: 1) requestor of exam; 2) diagnosis classification; and 3) immunohistochemical tests. Descriptive statistical analyses were done.

**Results:**

13,522 histopathological exams were performed, mean 1,229/year. The Public Health System of the city of Belo Horizonte was the main requestor of exams (77.13%), followed by private professionals (19.26%), and other cities (2.03%). Most lesions were considered benign (12,599/ 93.17%), with 854 malignant lesions (6.32%). 469 immunohistochemical tests were performed; 324 (69.08%) were from benign diagnosis, and 145 (30.92%) from malignant diagnosis. The most used antibodies were against S100, vimentin, smooth muscle actin, actin muscle specific HHF-35, and pan-cytokeratin AE1/AE3.

**Conclusions:**

Public Health System is the major user of the diagnostic service on oral pathology in our institution. Most diagnoses were of benign lesions, although many malignant lesions were detected. Immunohistochemistry was particularly important in solving challenging cases.

## Background

The most important Dental Schools in Brazil maintain oral pathology diagnostic laboratories, as occur in other countries [1]. At the Universidade Federal de Minas Gerais, which is the main public University of the State, and one of the most important Universities in Brazil, the Service of Oral and Maxillofacial Pathology was first established in 1966. From 1994 on, it is organized the way it is today. The laboratory’s main work is to perform histopathological and cytological exams of lesions affecting oral and maxillofacial tissues. Several health services send materials for analyses. Some of them comprise the Public Health System (PHS) of the city of Belo Horizonte, which includes all the dental clinics of the School of Dentistry of the Universidade Federal de Minas Gerais, the Clinics Hospital of the same University, the Odilon Behrens Municipal Hospital, among others. Another source for the specimens are the dental clinics of the PHS of other cities within the Minas Gerais State. Finally, private professionals also request exams. The oral pathologists working in the laboratory are professors at the School, and they are also coordinators of the service. They are licensed as specialists by the Regional Council of Dentistry and are fellows of the Brazilian Society of Oral Stomatology and Pathology (SOBEP). Master and doctorate students participate in the diagnostic process as well.

As stated before [1], providing such a service, the oral pathology service accomplishes four goals: to provide a diagnostic laboratory for the licensed practitioner and dental students; to obtain material that can be used for teaching purposes; to help training new oral pathologists; and to provide a source of research material.

Despite the professional and academic relevance of the Brazilian oral pathology diagnostic laboratories, no information about their user’s profile is available in the English literature, as there is for some services worldwide [1-5]. Thus, the aim of the present study was to investigate the profile of usage of an oral pathology diagnostic center over a 10-year period. We report data about the histopathological and immunohistochemical exams, as well as the main users of this regional reference laboratory. The findings reported herein should be relevant to compare with international reports. Moreover, the coordinators of the service can look for better strategies to improve the coverage of the service. Finally, we hope to contribute to the improvement of the oral pathology and oral medicine visibility among dentists, general pathologists, and medical staff.

## Methods

This retrospective study was performed at the Oral and Maxillofacial Pathology Service of the School of Dentistry of Universidade Federal de Minas Gerais. This is the Oral Pathology reference center in the state of Minas Gerais, which capital is the city of Belo Horizonte. Information about all histopathological exams performed between 2002 and 2012 was retrieved from the electronic files of the Service. These electronic files contain a database created within Microsoft Access software in 1997 and contain all biopsy records of the service since 2002.

Data collected for the study were saved into a database and included: 1) requestor of exam (PHS of Belo Horizonte; PHS of other cities in the State of Minas Gerais; or private professionals); 2) diagnosis classification (benign or malignant lesion); 3) immunohistochemical tests (performed or not, and for which antigen). Descriptive statistical analyses were done. No confidence intervals were calculated because this study is a census. The study was submitted to the Committee of Ethics in Research of the Universidade Federal de Minas Gerais (protocol number 30318914.2.0000.5149) and is in compliance with the Helsinki Declaration.

## Results

**Figure 1:**
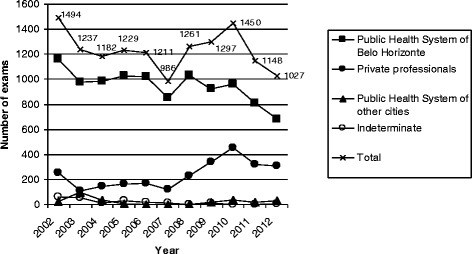
**Requestors of the exams over 10 years – absolute number.**

Over the 10 years, 13,522 histopathological exams were performed (Table 1). The mean number of exams in a year was 1,229. Most exams (1,494/11.05%) were done in 2002, followed by 2010 (1,450/10.72%) (Figure 1). The fewest number of exams was done in 2007 (986/7.29%), followed by 2012 (1,027/7.60%) (Figure 1).

**Table 1:** **Exams, requestors, diagnosis, and immunohistochemical tests performed in the laboratory from 2002 to 2012**

***Year***	***Requestors of the exams***								***Total***		***Diagnoses classification***						***Immunohistochemical tests***		
	***Public Health system of the city of Belo Horizonte***		***Private***		***Public Health system of other cities***		***Indeterminate***				***Benign***		***Malignant***		***Indeterminate***				
	***n***	***%***	***n***	***%***	***n***	***%***	***n***	***%***	***n***	***%***	***n***	***%***	***n***	***%***	***n***	***%***	***n***	%*	%**
2002	1,161	77.71	253	16.93	22	1.47	58	3.88	1,494	11.05	1,426	95.45	67	4.48	1	0.07	1	0.21	0.07
2003	980	79.22	107	8.65	97	7.84	53	4.28	1,237	9.15	1,167	94.34	64	5.17	6	0.49	28	5.97	2.26
2004	983	83.16	145	12.27	39	3.30	15	1.27	1,182	8.74	1,118	94.59	62	5.25	2	0.17	59	12.58	4.99
2005	1,025	83.40	165	13.43	9	0.73	30	2.44	1,229	9.09	1,158	94.22	71	5.78	0	0.00	28	5.97	2.28
2006	1,020	84.23	169	13.96	2	0.17	20	1.65	1,211	8.96	1,134	93.64	67	5.53	10	0.83	21	4.48	1.73
2007	854	86.61	118	11.97	3	0.30	11	1.12	986	7.29	894	90.67	68	6.90	24	2.43	44	9.38	4.46
2008	1,030	81.68	228	18.08	1	0.08	2	0.16	1,261	9.33	1,168	92.62	87	6.90	6	0.48	15	3.20	1.19
2009	925	71.32	339	26.14	18	1.39	15	1.16	1,297	9.59	1,232	94.99	65	5.01	0	0.00	75	15.99	5.78
2010	958	66.07	455	31.38	34	2.34	3	0.21	1,450	10.72	1,327	91.52	121	8.34	2	0.14	62	13.22	4.28
2011	809	70.47	318	27.70	20	1.74	1	0.09	1,148	8.49	1,058	92.16	87	7.58	3	0.26	68	14.50	5.92
2012	685	66.70	307	29.89	30	2.92	5	0.49	1,027	7.60	917	89.29	95	9.25	15	1.46	68	14.50	6.62
Total	10,430	77.13	2,604	19.26	275	2.03	213	1.58	13,522	100.00	12,599	93.17	854	6.32	69	0.51	469	100.00	3.47

%* = percentage of immunohistochemical tests per total of immunohistochemical tests performed over 10 years.

%** = percentage of immunohistochemical tests per total of exams performed in the same year.

**Figure 2:**
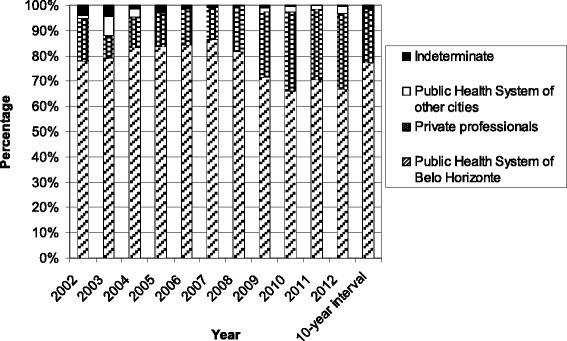
**Requestors of the exams over 10 years - percentage.**

The PHS of Belo Horizonte was the main requestor of exams in all years, accounting for 77.13% of the total of exams over the 10 years (ranging from 66.07% to 86.61%) (Table 1, Figures 1 and 2). The PHS of other cities requested 2.03% of exams (ranging from 0.09% to 4.28%, Table 1, Figures 1 and 2). Private professionals sent 19.26% of exams over 10 years (ranging from 8.65% to 31.38%) (Table 1, Figures 1 and 2).

Most lesions were considered benign (12,599/93.17%), with 854 malignant lesions (6.32%), and 69 (0.51%) indeterminate diagnoses. In 2010, we had the highest number of malignant lesions (121/8.34%); nevertheless, it was in 2012 that these lesions represented the highest percentage of diagnosis (95/9.25%) (Table 1). Of interest, the percentage of malignant lesions diagnosed in specimens sent by the PHS of Belo Horizonte (6.91%) was slightly higher than the mean, followed by requests from PHS of other cities (4.49%), and private professionals (5.09%) (Table 2). An indeterminate diagnosis was mostly established for lesions from PHS of other cities (1.09%), followed by PHS of Belo Horizonte (0.41%) and private practitioners (0.19%) (Table 2).

**Table 2:** **Diagnosis of lesions from each requestor, over 10 years**

***Requestor***	***Diagnosis***							
	***Benign***		***Malignant***		***Indeterminate***		***Total***	
	***n***		***n***		***n***		***n***	
Public Health System of Belo Horizonte	9,666	92.67	721	6.91	43	0.41	10,430	77.13
Private professionals	2,482	95.31	117	4.49	5	0.19	2,604	19.26
Public Health System from other cities	258	93.82	14	5.09	3	1.09	275	2.03
Indeterminate	193	90.61	2	0.94	18	8.45	213	1.58
Total	12,599	93.17	854	6.32	69	0.51	13,522	100.00

**Figure 3:**
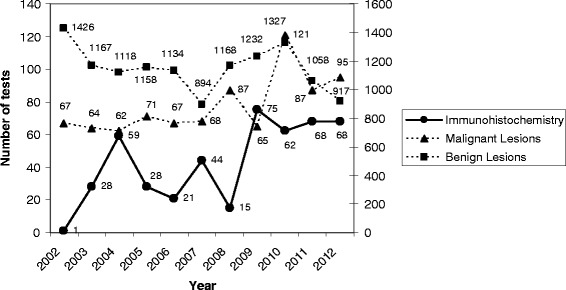
**Absolute number of immunohistochemical tests and diagnoses of benign and malignant lesions.** The main axis refers to “immunohistochemistry” and “malignant lesions” values. The secondary axis refers to “benign lesions” values.

In total, 469 immunohistochemical tests were performed, with varying amounts over years (Table 2, Figure 3). During the whole period, 324 (69.08%) immunohistochemical tests were from benign diagnoses, and 145 (30.92%) from malignant diagnoses. Most immunohistochemical tests 369 (78.68%) were performed in specimens sent by the PHS of Belo Horizonte, followed by 98 (20.89%) from private professionals, and 2 (0.43%) from PHS of other cities.

**Figure 4:**
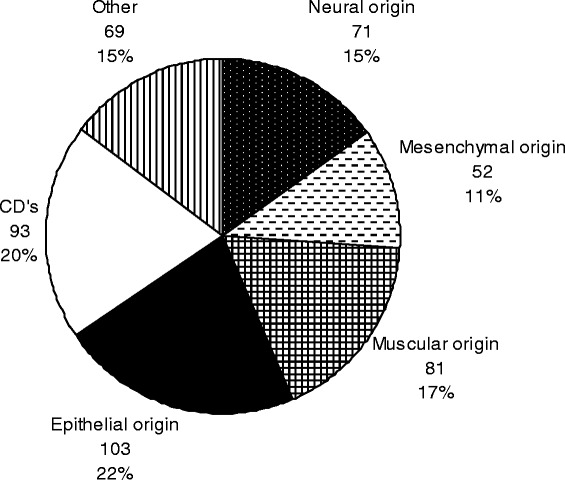
**Antibodies’ specificity used in immunohistochemistry.**

Forty-five different antibodies were used in immunohistochemical tests (Table 3). The most used ones were against S100 (65/13.86%), vimentin (51/10.87%), smooth muscle actin (34/7.24%), actin muscle specific HHF-35 (31/6.60%), and AE1/AE3 (28/5.97%). Figure 4 shows the types of antibodies used, grouped according to their specificity.

**Table 3:** **Specificity and amount of the immunohistochemical tests performed over 10 years**

***Antigen***	Number of tests*
S100	65
Vimentin	51
Smooth muscle actin	34
Actin muscle specific (HHF-35)	31
Pan-cytokeratin (AE1/AE3)	28
CK19	18
CD20, CD34	17
CD3	15
Desmin, CK7, Ki-67	14
CK13, CK14	13
CD68, Bcl-2	11
CK8	9
Epithelial membrane antigen, Plasmacell	7
P53, Leucocyte common antigen, CD1a, Glut-1, CD99	5
CD45RO, Lambda, Kappa, CD10, CD5	4
CD31, CD56, Enolase neuron-specific, CD79, Glial fibrilary acidic protein	3
Metallothionein	2
Laminin, Tryptase, c-ERB2, Calponin, Androgen, CK20, CD30, Factor 13a, CD57, Caldesmon	1
Indeterminate	8

*antibodies with the same number of tests are grouped.

## Discussion

The present study reports the profile of usage of a reference oral and maxillofacial pathology laboratory along 10 years. It is noticeable that the PHS of the city of Belo Horizonte accounted for most specimens sent to the laboratory all over the years. This System encompasses some Institutions, including the School of Dentistry where the laboratory is located. It should be taken into account that the Health Institutions linked to this System provide a free dental care in oral medicine and oral pathology, and many specialists in these areas work in those Institutions. This probably explains the high percentage of biopsies from PHS found in the study. Of relevance, some authors have already reported that general dentists usually refer the patients needing oral pathology evaluation and biopsy to a specialist [5]. This can be taking place in Belo Horizonte city as well, contributing for the centralization of dental care in oral pathology and oral medicine at the reference Institutions, *i.e.* the ones from the PHS.

Interestingly, along the 10 years, and especially from 2008 on, we could observe an increase in the specimens sent by private practitioners. In their 30-year retrospective, Franklin and Jones [3] also reported a fourfold increase in number of specimens received from general dentistry practitioners. This augmentation should be due to some factors. First, more specialists in Stomatology, oral pathology, and oral medicine should be working in their private offices, performing the biopsies in their own offices. Thus, a lower number of professionals are referring patients to the public health institutions to perform those procedures. Second, maybe general dentists are becoming more confident to perform oral biopsies in their daily practice [5]. Third, maybe private practitioners used to send their specimens to general pathology services, instead of to an oral pathology laboratory [2]. Along the years, the high quality work offered by the oral pathology service helped private practitioners to know and trust the service, thus they could send their specimens to us. Oral pathologists are more capable in diagnosing pathologic conditions which are more specific of their area of work, like salivary glands lesions and odontogenic tumors [2,6]. Interestingly, despite some dentists still refer to general pathologists, Barret and Speight [6] found that most (97.6%) general pathologists were aware of oral pathologists in United Kingdom, and 91.6% perceived a need for them.

Of relevance, when private practitioners send exams to our laboratory, a fee is charged for the service, while when the PHS is the requestor of the exam, no fee is applied and the services are subsidized by the government. Concerning this, Chugh et al. [2] reported that at the oral pathology diagnostic service of Faculty of Dentistry in Toronto, more than 90% of the specimens were from private practitioners, but there was a decrease of 31% in the number of specimens sent to the laboratory after the removal of a subsidy.

Malignant lesions comprised 6.32% of the lesions diagnosed over 10 years in the present study. It is interesting to observe that the percentage was similar among the three requestors. Nevertheless, considering the absolute number, the great majority of malignant lesions were from PHS. These data have an important epidemiological and financial impact for the PHS, which is responsible for offering the complete treatment for those patients. Apart of this, malignant lesions accounted for a median of 1.38% of diagnoses in the 4-year retrospective study reported by Chugh et al. [2]. Franklin and Jones [3] reported a 30-year retrospective study of specimens sent by general dental practitioners, with only 9 malignant tumors. Accordingly, during the year evaluated by Wan e Savage [5], all the 26 cases of malignancies received by their laboratory were from specialists. These findings seem to indicate that general dentists usually refer patients with suspicion of a malignant lesion for specialists [3].

Our data reveal that immunohistochemistry was applied in 3.47% of cases, either malignant or benign. This tool was important to establish diagnoses of malignant salivary gland neoplasms, mesenchymal lesions and others. The most useful markers were S100, vimentin, smooth muscle actin, actin muscle specific, and pan-cytokeratin. These antigens accounted for 44.6% of all reactions. This profile can help other laboratories to select which antibodies they should have to start an immunohistochemical service for diagnostic proposals.

At the Oral and Maxillofacial Pathology Service, immunohistochemistry has been an important tool in reaching the diagnosis of malignant salivary gland tumors. Diagnoses of those tumors are challenging given the histological overlap among various subtypes and their morphologic heterogeneity [7]. This fact also explains the most employed antibodies used in the service, markers to luminal (low-molecular weight cytokeratins), abluminal and myoepithelial cells (high-molecular cytokeratins), and against myoid proteins, besides other driven specifically to myoepithelial cells (Figure 4).

Moreover, the vast majority of diagnoses could be performed within the microscopic evaluation of routinely stained material only. Instead, immunohistochemistry should be useful to confirm diagnostic hypothesis in some cases, helping to identify some rare or atypical benign lesions, or to characterize malignant tumors [8]. Both situations are exemplified in our service with diagnoses of lesions such as solitary fibrous tumor [9] and lymphomas [10] (Figure 4).

## Conclusions

The present study is the first one in English literature to report an evaluation of the profile of usage of a Brazilian oral pathology service. The Public Health System of the city of Belo Horizonte is responsible for the majority of exams. An increase in the number of exams from particular practitioners was observed. Most diagnoses performed over 10 years were benign, although many malignant lesions were detected as well. Immunohistochemistry proved to be useful in some cases, either malignant or benign. S100, vimentin, smooth muscle actin, actin muscle specific, and pan-cytokeratin antigens were the most used ones.

This study emphasizes that it is indispensable to submit every tissue removed from the oral cavity and maxillofacial apparatus to histopathological analysis. This reflects an increasing need for oral pathology services. Studies on this issue should be encouraged to contribute to the improvement of the oral pathology and oral medicine visibility, among dentists, general pathologists, or medical staff.

## Abbreviations

PHS: Public Health System
SOBEP: Brazilian Society of Oral Stomatology and Pathology
